# Case-based learning: a case of maturity-onset diabetes of the young 5 (MODY5) due to 17q12 microdeletion with a diminished plasma glucagon level

**DOI:** 10.1007/s13340-025-00804-2

**Published:** 2025-02-17

**Authors:** Yoko Sugano, Motohiro Sekiya, Yuki Murayama, Yoshinori Osaki, Hitoshi Iwasaki, Hiroaki Suzuki, Hiroko Fukushima, Hisato Suzuki, Emiko Noguchi, Hitoshi Shimano

**Affiliations:** 1 Department of Endocrinology and Metabolism, Institute of Medicine, University of Tsukuba, 1-1-1 Tennodai, Tsukuba, Ibaraki 305-8575 Japan; 2 Department of Child Health, Institute of Medicine, University of Tsukuba, Tsukuba, Japan; 3 Department of Medical Genetics, Institute of Medicine, University of Tsukuba, Tsukuba, Japan

**Keywords:** MODY5, HNF1B, 17q12 microdeletion, Glucagon

## Abstract

Maturity-onset diabetes of the young type 5 (MODY5), causally associated with loss-of-function of the *HNF1B* gene, is a rare form of monogenic diabetes that has been underdiagnosed in part because microdeletions of chromosome 17q12 encompassing the *HNF1B* gene cannot be detected by sequencing-based approaches, which accounts for about 50% of MODY5 cases. We herein describe a 37-year-old Japanese woman who manifested diabetic ketosis at the onset. The coexistence of features associated with MODY5, including abnormal renal function, impaired insulin secretion, pancreatic hypoplasia and hypomagnesemia, prompted us to decode her genomic information using whole-exome sequencing, where we were not able to identify any pathogenic *HNF1B* gene mutations. We further examined her genomic integrity using multiplex ligation probe amplification (MLPA) analysis, leading to identification of the 17q12 microdeletion which was further supported by array comparative genomic hybridization (array-CGH). Her insulin secretory capacity was insufficient, whereas her total daily dose of insulin was 11 U/day (0.25 U/Kg/day), indicating that she was relatively sensitive to insulin. As a possible explanation, we found that her plasma glucagon level was below the detection limit. Since inactivation of acetyl-CoA carboxylase 1 (*ACACA*), encoded in close proximity to the *HNF1B* gene, was reported to blunt glucagon secretion, the concurrent deletion of the *ACACA* gene may be in part responsible for this manifestation. In conclusion, the genetic analyses of MODY5 cases require the judicious use of appropriate genetic technologies. In addition, alpha-cell dysfunction may at least in part account for the variable clinical manifestations of MODY5.

**Supplementary Information:**

The online version contains supplementary material available at 10.1007/s13340-025-00804-2.

## Introduction

Maturity-onset diabetes of the young type 5 (MODY5) is one of the monogenic forms of diabetes [1, 2] and is caused by haploinsufficiency of the *HNF1B* gene [3]. While it is widely recognized that MODY5 is frequently accompanied by renal manifestations, the *HNF1B*-related syndrome encompasses a wide spectrum of abnormalities, including pancreas hypoplasia, hypomagnesemia, facial dysmorphism and neurodevelopmental disorders [2, 4]. Unlike other monogenic diabetes, about half of MODY5 cases are associated with a 17q12 deletion spanning approximately 1.5 Mb with variations from case to case encompassing 15 genes on average [4, 5], including *HNF1B* [6], which explains the relatively high prevalence of sporadic cases.

The microdeletions cannot be detected by sequencing-based approaches, including whole-exome sequencing, and the inherent limitations of these methodologies pose a challenge in the diagnostic process of MODY5. Several approaches are available for detecting deletions or duplications with recent technical advances. Among them, multiplex ligation-dependent probe amplification (MLPA) is a state-of-the-art technique where MLPA probes hybridized to the sample DNA are amplified and the copy number changes can be quantified [7]. Array comparative genomic hybridization (array-CGH) is another technique for genome-wide screening of segmental genomic alterations: DNA samples from a control and a patient are differentially labeled and the gains or losses of copy numbers can be inferred through the competitive hybridization against a normal chromosome spread [8].

In this report, we describe a case of MODY5 with 17q12 microdeletion diagnosed with these two technologies. Intriguingly, the patient manifested impaired secretion of both insulin and glucagon.

## Materials and methods

### Genetic analysis

DNA extraction from blood peripheral mononuclear neutrophils and whole-exome-sequencing analysis were performed as described previously [9]. The 17q12 microdeletion was detected by the MLPA method with the MODY Mix-2 probe set (P357, Falco Biosystems). Two probes for each gene of interest were designed to be complementary to the immediately adjacent sequences of targeted regions. After hybridization of the paired probes, the probes were enzymatically ligated and amplified by the common PCR primer sequences with fluorescent labeling included in the oligonucleotide probes. The PCR products were separated by capillary electrophoresis, and the allelic copy number was inferred based on the corresponding peaks of PCR product [7]. To ensure the accuracy of the analysis, we recruited three healthy volunteers for control subjects. The sequence data were analyzed by Coffalyser. Net software (MRC-Holland, Netherlands, version 210,604.1451).

The array-CGH was performed using the service of SRL (the Center for Molecular Biology and Cytogenetics Gene Analysis Section, Hino, Tokyo, Japan). The hybridization was carried out using GenetiSure Dx Postnatal Assay (Agilent Technologies) following their instructions. The patient’s DNAs and gender-matched control DNAs were enzymatically digested and differentially labeled with Cy5 and Cy3. After hybridization and purification, the array was scanned using a SureScan Dx Microarray scanner (G5761A, Agilent Technologies).

### Glucagon measurement

We measured plasma glucagon levels using an ELISA system (Cosmic Corporation Co., Ltd., Tokyo, Japan) with optimal sensitivity and selectivity [10].

## Case report

We herein describe a 37-year-old Japanese woman who manifested diabetic ketosis at the onset (17 years old). The coexistence of typical features associated with MODY5 including renal cysts, impaired insulin secretion, pancreatic hypoplasia and hypomagnesemia, implicated genetic defect(s) in the *HNF1B* gene.

**Fig. 1:**
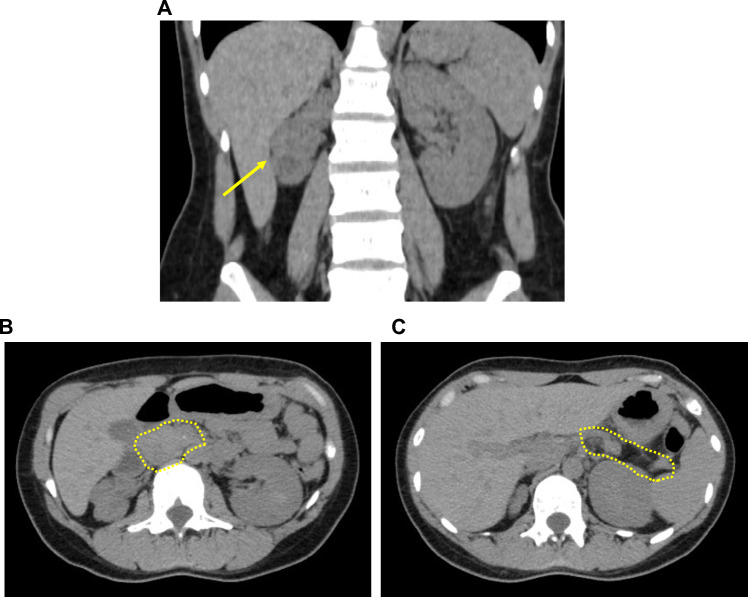
Morphological assessment of the patient’s kidneys and pancreas by CT scanning. **A**. Both kidneys captured in a coronal section. A yellow arrow indicates the atrophic right kidney. **B, C**. The agenesis of distal pancreas. The pancreatic head can be captured in a horizontal section (B, indicated by a yellow dotted circle) but the dorsal pancreas cannot be captured in sections with the spleen (the lack of pancreas is indicated by a yellow dotted circle)

At the age of 16 years, her annual health check-up detected mild renal dysfunction (Ccr 77.3 ml/min, urinary protein 0.25 g/day). At the age of 17 years, she was hospitalized due to elevated blood glucose levels with ketosis (blood glucose 29.2 mmol/l, HbA1c 17.2%, 3-hydroxybutyrate 6912 μmol/l). On admission, her body mass index (BMI) was 17.9 (height: 150 cm, weight: 40 kg). After hydration and insulin treatment, we conducted a comprehensive evaluation of the patient to identify the factors and parameters causally linked to the development of her diabetes. While her insulin secretory capacity was impaired based on our biochemical analyses (fasting blood glucose 12.8 mmol/l, serum C-peptide 0.7 ng/ml, urinary C-peptide excretion 14.9 μg/day), all the autoantibodies examined, glutamate decarboxylase (GAD), insulinoma-associated protein-2 (IA-2) and islet cell antibody (ICA), were negative. We also examined her renal function by standard renography where we found that only the right kidney was dysfunctional with excretion delay (renal plasma flow: total 353 ml/min, right: 55 ml/min, left: 298 ml/min). CT-imaging-based assessment of renal morphology revealed atrophy with multiple cysts, specifically in the right kidney, consistent with the functional test results (Fig. 1A). We also found agenesis of the dorsal pancreas which lacked the pancreatic body and tail (Fig. 1B, C). In addition, she had hypomagnesemia (1.4 mg/dl) with inappropriately increased urinary excretion of magnesium (FEMg 9.9%). She had hypokalemia (2.8 mEq/l), possibly secondary to the hypomagnesemia. In our physical examination, facial deformities such as depressed nasal bridge, deep set eyes and down-slanting palpebral fissures could also be appreciated.

We managed these conditions by insulin injection and magnesium supplementation. Since discharge, her blood glucose levels were well controlled (HbA1c ~ 6.9%) with a relatively small amount of insulin (total daily dose ~ 11 U/day) for a long time. We re-evaluated her insulin secretory capacity 20 years after onset by measuring fasting blood glucose level as well as serum C-peptide (blood glucose: 5.6 mmol/l, C-peptide 0.4 ng/ml), which were unchanged compared to at onset. These observations suggest that she had been insulin-sensitive in the presence of impaired insulin secretion. As a first step, we measured plasma glucagon in the same blood sample using an ELISA system with improved sensitivity and specificity [10]. Unexpectedly her glucagon level was below the detection limit, even in the presence of normoglycemia.

We next surveyed the penetrance and heritability of these traits. Although her parents and siblings were all unaffected, she has a 7-year-old son who shares some of these traits: polycystic kidney, slightly elevated serum creatinine level (0.25 mg/dl at 1.5 years of age) and hypomagnesemia (1.5 mg/dl at 2 years of age). Although she did not have any signs and symptoms of intellectual disabilities, her son was diagnosed with attention deficit hyperactivity disorder. She appeared to be the proband carrying de novo mutation(s) that can be dominantly inherited to offspring (Supplementary Fig. 1).

**Fig. 2:**
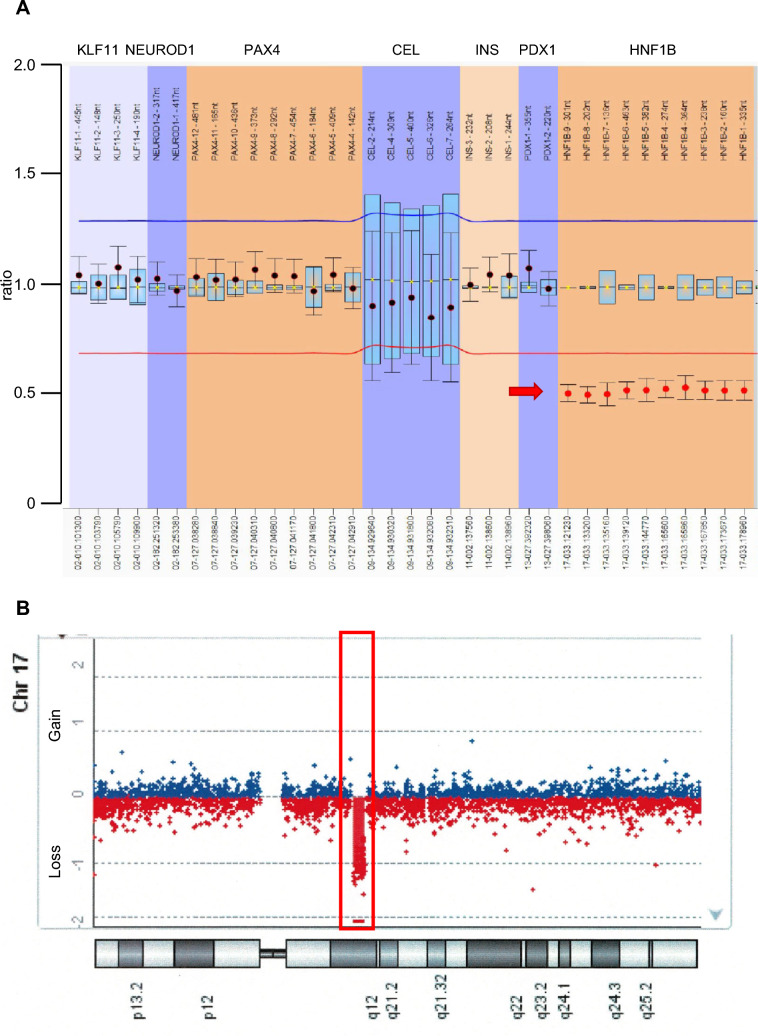
Genetic analyses to identify the 17q12 microdeletion. **A**. MLPA analysis. The ratio was calculated based on the peak area compared to the control subjects for each probe. The red arrow indicates the hemizygous deletion of *HNF1B* gene in this case. **B**. Array-CGH result along with chromosome 17. Each dot represents a gain or a loss of one oligonucleotide probe and its position on the chromosome. The 17q12 microdeletion identified in this case is highlighted with a red rectangle

Based on the pervasive impacts of the potential genetic defect(s) on multiple tissues and cells, we first attempted to decipher her genomic information by unbiased whole-exome sequencing analysis. However, we were not able to find any pathogenic mutations in either *HNF1B* or other genes reported to be associated with monogenic diabetes [11]. Therefore, we examined the integrity of the genomic loci encompassing the *HNF1B* gene using MLPA analysis, where we identified a hemizygous 17q12 microdeletion (Fig. 2A, Supplementary Table 1). Since we were able to detect genomic deletion with this analysis, we further took advantage of array-CGH to determine the genomic region(s) affected in this case at genome-wide scale (Fig. 2B). This subsequent validation not only corroborated the observed genetic deletion at the 17q12 but also determined the deleted region spanning about 1.4 Mb in size [GRCh37/hg19 NC_000017.12..(34822500–36248918)_(34801811–36290256)del] that encompasses genes including *ZNHIT3*, *MYO19*, *PIGW*, *GGNBP2*, *DHRS11*, *MRM1*, *LHX1*, *AATF*, *ACACA*, *TADA2A*, *DUSP14*, *SYNRG*, *DDX52*, *HNF1B* and *YWHAEP7*. Although genetic analysis of her son has not been performed due to ethical limitations, the 17q12 microdeletion would sufficiently explain the aforementioned clinical manifestations observed in her family.

## Discussion

We identified a 17q12 microdeletion in a patient allowing the diagnosis of MODY5. The diagnosis of MODY5 exclusively relies on genetic analyses that are not available in our routine clinical practice, and about half of cases can be explained by 17q12 microdeletions [4, 5] that cannot be detected by sequencing-based approaches. Because of these technical difficulties, MODY5 has been underdiagnosed and we need to accumulate evidence to establish diagnostic algorithms and improve therapeutic approaches. Recent advancements in massively parallel sequencing have enabled us to identify pathogenic mutations responsible for a wide variety of diseases. However, we need to recognize both the promises and pitfalls of this technique. As we have demonstrated in this case, we cannot identify deletions of genomic loci with this approach. Furthermore, the presence of pseudogenes can hamper exome-based diagnosis, as demonstrated in the case of by 21-hydroxylase deficiency [12].

The detailed genotype–phenotype correlation in MODY5 cases awaits further investigation [1, 4]. The clinical manifestations in cases of *HNF1B* mutations may exhibit differential patterns depending on the functional domains affected by the mutations, considering the complex regulation of *HNF1B* transcriptional activities as exemplified by interactions with other proteins [13]. In cases of 17q12 microdeletions, genomic loci spanning 1.5 Mb on average are deleted that encode genes such as *AATF*, *ACACA*, *C17orf78*, *DDX52*, *DHRS11*, *DUSP14*, *GGNBP2*, *HNF1B*, *LHX1*, *MRM1*, *MYO19*, *PIGW*, *SYNRG*, *TADA2A* and *ANHIT3* [14]. The phenotypic features [14, 15] may vary among individuals depending on the influenced regions. It has been reported that insulin secretory capacities and ages of onset in MODY5 cases vary among individuals [1, 2]. However, these observations have been discussed mainly based on the requirement of insulin therapy that reflects both insulin secretion and insulin sensitivity. Intriguingly, our case showed a markedly decreased serum glucagon level that would have significantly contributed to the relatively low daily dose of insulin. While counter regulatory hormones have been often dismissed in the analyses of MODY5, our case suggests that we need to pay more careful attention to these hormones. Notably, it was recently reported that deficiency of acetyl-CoA carboxylase 1 (ACC1) encoded by the *ACACA* gene blunts glucagon secretion [16]. Since the *ACACA* gene is encoded in close proximity of the *HNF1B* gene and is centrally located in the commonly deleted regions in the 17q12 syndrome [14], 17q12 microdeletions would delete the *ACACA* gene together with *HNF1B*. Although potential coexistence of other mutations may be responsible for the decreased plasma glucagon concentration in this case, we were not able to find any pathogenic nonsynonymous mutations in proglucagon gene that encodes glucagon, the processing enzyme (i.e., prohormone convertase 2, PC2) or other genes related to alpha-cell functions. She has haploinsufficiency of ACACA gene since our exome-sequencing survey did not reveal any pathogenic mutations in the ACACA gene in the intact allele. Our observation is in sharp contrast to the increased glucagon concentrations in diabetic patients [17], as well as the fact that glucagon secretion is increased in the absence of insulin-mediated suppression [18]. The S148L mutation in *HNF1B* was also reported to be associated with increased glucagon secretion with compensatory mechanisms [19]. These observations implicate that the 17q12 deletion may specifically decrease plasma glucagon concentrations. Whereas the glucagon level in a case of 17q12 deletion recently reported was not affected, the discrepancy may arise because of the different ELISA systems employed, since the specificity of glucagon detection systems has been a matter of debate [20], or because of differences in the genomic regions affected [21]. Despite this uncertainty, this perspective deserves further scrutiny to better understand glucose homeostasis in MODY5 cases. The therapeutic potential of inhibition of glucagon’s action in type 1 diabetes has been extensively investigated [22, 23], and a phase II trial to examine the effects of the glucagon receptor antagonist volagidemab was reported recently [24]. Our case can also be a piece of human data to facilitate our understanding of this perspective. The potential contributions of *ACACA* gene deletion to the renal phenotypes in MODY5 have been reported [1], which was recently supported by a rodent study [25] albeit with some debates regarding whether ACC1 alone suffices or not [26]. In other words, MODY5 cases with relatively preserved renal functions may carry a 17q12 microdeletion rather than mutations in the *HNF1B* gene, and we may be able to choose our genetic approaches based on the clinical course of renal functions of patients although the controversies need to be clarified experimentally and clinically in the future.

It is also important to acknowledge the limitations in this study. Although we surveyed her genome using the exome-sequencing approach as well as technologies to detect microdeletions, we may need to consider potential contributions of mutations in regulatory regions such as promoters and introns that were not covered by our approaches in this study [27]. In addition, we assumed that the diminished glucagon would be at least in part responsible for the enhanced insulin sensitivity. However, MODY5 patients especially with microdeletion tend to be leaner [1], which may be responsible for her insulin sensitivity. Moreover, we were not able to quantify her insulin sensitivity with historically established approaches (i.e., homeostasis model assessment of insulin resistance (HOMA-IR), Matsuda index, insulin sensitivity factor or the glucose clamp technique), which could be another limitation for us to compare our case with others reported in the literature. Lastly, another issue remains to be solved is the question whether or not the haploinsufficiency of ACACA gene is sufficient to cause the diminished glucagon secretion observed in our case. The alpha-cell-specific ACACA knockout mice exhibited some residual mRNA expression of ACACA gene, and neither gene dose effects nor different concentrations of the ACC inhibitor was tested in the preceding report [16]. Therefore, this question needs to be addressed by careful studies in the future. However, we would speculate that there might be additional unidentified defects in our case to reconcile all previous reports including ours to ensure consistency.

In conclusion, we diagnosed a case of MODY5 carrying a 17q12 microdeletion. When we encounter patients with clinical features consistent with MODY5, the potential pitfalls in exome sequencing should be kept in mind. Furthermore, we would like to emphasize that more attention may need to be paid to glucagon, since MODY5 is prejudiced to be a disease with impaired insulin secretion in a simplified view.

## Supplementary Information

Below is the link to the electronic supplementary material.

Supplementary file1 (PPTX 46 KB)

Supplementary file2 (XLSX 12 KB)

## References

[1] Dubois-Laforgue D, Cornu E, Saint-Martin C, Coste J, Bellanné-Chantelot C, Timsit J. Diabetes, associated clinical spectrum, long-term prognosis, and genotype/phenotype correlations in 201 adult patients with hepatocyte nuclear factor 1B (HNF1B) molecular defects. Diabetes Care. 2017;40(11):1436–43. 10.2337/dc16-2462. 28420700 10.2337/dc16-2462

[2] Horikawa Y. Maturity-onset diabetes of the young as a model for elucidating the multifactorial origin of type 2 diabetes mellitus. J Diabet Investig. 2018;9(4):704–12. 10.1111/jdi.12812. 10.1111/jdi.12812 PMC6031504 29406598

[3] Horikawa Y, Iwasaki N, Hara M, Furuta H, Hinokio Y, Cockburn BN, et al. Mutation in hepatocyte nuclear factor-1 beta gene (TCF2) associated with MODY. Nat Genet. 1997;17(4):384–5. 10.1038/ng1297-384. 9398836 10.1038/ng1297-384

[4] Clissold RL, Hamilton AJ, Hattersley AT, Ellard S, Bingham C. HNF1B-associated renal and extra-renal disease-an expanding clinical spectrum. Nat Rev Nephrol. 2015;11(2):102–12. 10.1038/nrneph.2014.232. 25536396 10.1038/nrneph.2014.232

[5] Horikawa Y, Enya M, Fushimi N, Fushimi Y, Takeda J. Screening of diabetes of youth for hepatocyte nuclear factor 1 mutations: clinical phenotype of HNF1β-related maturity-onset diabetes of the young and HNF1α-related maturity-onset diabetes of the young in Japanese. Diabet Med. 2014;31(6):721–7. 10.1111/dme.12416. 24905847 10.1111/dme.12416

[6] Bellanné-Chantelot C, Clauin S, Chauveau D, Collin P, Daumont M, Douillard C, et al. Large genomic rearrangements in the hepatocyte nuclear factor-1beta (TCF2) gene are the most frequent cause of maturity-onset diabetes of the young type 5. Diabetes. 2005;54(11):3126–32. 10.2337/diabetes.54.11.3126. 16249435 10.2337/diabetes.54.11.3126

[7] Sellner LN, Taylor GR. MLPA and MAPH: new techniques for detection of gene deletions. Hum Mutat. 2004;23(5):413–9. 10.1002/humu.20035. 15108271 10.1002/humu.20035

[8] Lockwood WW, Chari R, Chi B, Lam WL. Recent advances in array comparative genomic hybridization technologies and their applications in human genetics. Eur J Hum Genet EJHG. 2006;14(2):139–48. 10.1038/sj.ejhg.5201531. 16288307 10.1038/sj.ejhg.5201531

[9] Sekiya M, Matsuda T, Yamamoto Y, Furuta Y, Ohyama M, Murayama Y, et al. Deciphering genetic signatures by whole exome sequencing in a case of co-prevalence of severe renal hypouricemia and diabetes with impaired insulin secretion. BMC Med Genet. 2020;21(1):91. 10.1186/s12881-020-01031-z. 32375679 10.1186/s12881-020-01031-z PMC7201978

[10] Matsuo T, Miyagawa J, Kusunoki Y, Miuchi M, Ikawa T, Akagami T, et al. Postabsorptive hyperglucagonemia in patients with type 2 diabetes mellitus analyzed with a novel enzyme-linked immunosorbent assay. J Diabet Investig. 2016;7(3):324–31. 10.1111/jdi.12400. 10.1111/jdi.12400 PMC4847885 27330717

[11] Murphy R, Ellard S, Hattersley AT. Clinical implications of a molecular genetic classification of monogenic beta-cell diabetes. Nat Clin Pract Endocrinol Metab. 2008;4(4):200–13. 10.1038/ncpendmet0778. 18301398 10.1038/ncpendmet0778

[12] New MI, Abraham M, Gonzalez B, Dumic M, Razzaghy-Azar M, Chitayat D, et al. Genotype-phenotype correlation in 1,507 families with congenital adrenal hyperplasia owing to 21-hydroxylase deficiency. Proc Natl Acad Sci USA. 2013;110(7):2611–6. 10.1073/pnas.1300057110. 23359698 10.1073/pnas.1300057110 PMC3574953

[13] Sneha P, Thirumal Kumar D, Lijo J, Megha M, Siva R, George P, Doss C. Probing the protein-protein interaction network of proteins causing maturity onset diabetes of the young. Adv Protein Chem Struct Biol. 2018;110:167–202. 10.1016/bs.apcsb.2017.07.004. 29412996 10.1016/bs.apcsb.2017.07.004

[14] Roberts JL, Gandomi SK, Parra M, Lu I, Gau CL, Dasouki M, et al. Clinical report of a 17q12 microdeletion with additionally unreported clinical features. Case Rep Genet. 2014;2014: 264947. 10.1155/2014/264947. 24991439 10.1155/2014/264947 PMC4060289

[15] Roehlen N, Hilger H, Stock F, Gläser B, Guhl J, Schmitt-Graeff A, et al. 17q12 deletion syndrome as a rare cause for diabetes mellitus type MODY5. J Clin Endocrinol Metab. 2018;103(10):3601–10. 10.1210/jc.2018-00955. 30032214 10.1210/jc.2018-00955

[16] Veprik A, Denwood G, Liu D, Bany Bakar R, Morfin V, McHugh K, et al. Acetyl-CoA-carboxylase 1 (ACC1) plays a critical role in glucagon secretion. Commun Biol. 2022;5(1):238. 10.1038/s42003-022-03170-w. 35304577 10.1038/s42003-022-03170-w PMC8933412

[17] Dunning BE, Foley JE, Ahrén B. Alpha cell function in health and disease: influence of glucagon-like peptide-1. Diabetologia. 2005;48(9):1700–13. 10.1007/s00125-005-1878-0. 16132964 10.1007/s00125-005-1878-0

[18] Kawamori D, Kurpad AJ, Hu J, Liew CW, Shih JL, Ford EL, et al. Insulin signaling in alpha cells modulates glucagon secretion in vivo. Cell Metab. 2009;9(4):350–61. 10.1016/j.cmet.2009.02.007. 19356716 10.1016/j.cmet.2009.02.007 PMC2694613

[19] Teo AK, Lau HH, Valdez IA, Dirice E, Tjora E, Raeder H, et al. Early developmental perturbations in a human stem cell model of MODY5/HNF1B pancreatic hypoplasia. Stem cell Rep. 2016;6(3):357–67. 10.1016/j.stemcr.2016.01.007. 10.1016/j.stemcr.2016.01.007 PMC4788763 26876668

[20] Kobayashi M, Maruyama N, Yamamoto Y, Togawa T, Ida T, Yoshida M, et al. A newly developed glucagon sandwich ELISA is useful for more accurate glucagon evaluation than the currently used sandwich ELISA in subjects with elevated plasma proglucagon-derived peptide levels. J Diabet Investig. 2023;14(5):648–58. 10.1111/jdi.13986. 10.1111/jdi.13986 PMC10119918 36729958

[21] Omura Y, Yagi K, Honoki H, Iwata M, Enkaku A, Takikawa A, et al. Clinical manifestations of a sporadic maturity-onset diabetes of the young (MODY) 5 with a whole deletion of HNF1B based on 17q12 microdeletion. Endocr J. 2019;66(12):1113–6. 10.1507/endocrj.EJ19-0020. 31391355 10.1507/endocrj.EJ19-0020

[22] Wang MY, Yan H, Shi Z, Evans MR, Yu X, Lee Y, et al. Glucagon receptor antibody completely suppresses type 1 diabetes phenotype without insulin by disrupting a novel diabetogenic pathway. Proc Natl Acad Sci USA. 2015;112(8):2503–8. 10.1073/pnas.1424934112. 25675519 10.1073/pnas.1424934112 PMC4345619

[23] Wang MY, Dean ED, Quittner-Strom E, Zhu Y, Chowdhury KH, Zhang Z, et al. Glucagon blockade restores functional β-cell mass in type 1 diabetic mice and enhances function of human islets. Proc Natl Acad Sci USA. 2021;118:9. 10.1073/pnas.2022142118. 10.1073/pnas.2022142118 PMC7936318 33619103

[24] Pettus J, Boeder SC, Christiansen MP, Denham DS, Bailey TS, Akturk HK, et al. Glucagon receptor antagonist volagidemab in type 1 diabetes: a 12-week, randomized, double-blind, phase 2 trial. Nat Med. 2022;28(10):2092–9. 10.1038/s41591-022-02011-x. 36192552 10.1038/s41591-022-02011-x PMC9872851

[25] Harley G, Katerelos M, Gleich K, de Souza DP, Narayana VK, Kemp BE, et al. Blocking AMPK signalling to acetyl-CoA carboxylase increases cisplatin-induced acute kidney injury and suppresses the benefit of metformin. Biomed Pharmacother. 2022;153: 113377. 10.1016/j.biopha.2022.113377. 36076520 10.1016/j.biopha.2022.113377

[26] Kampe K, Sieber J, Orellana JM, Mundel P, Jehle AW. Susceptibility of podocytes to palmitic acid is regulated by fatty acid oxidation and inversely depends on acetyl-CoA carboxylases 1 and 2. Am J Physiol Renal Physiol. 2014;306(4):F401–9. 10.1152/ajprenal.00454.2013. 24338821 10.1152/ajprenal.00454.2013 PMC3920022

[27] Kuwabara-Ohmura Y, Iizuka K, Liu Y, Takao K, Nonomura K, Kato T, et al. A case of MODY5-like manifestations without mutations or deletions in coding and minimal promoter regions of the HNF1B gene. Endocr J. 2020;67(9):981–8. 10.1507/endocrj.EJ20-0038. 32461507 10.1507/endocrj.EJ20-0038

