# Supplementary material for: Case-based learning: a case of maturity-onset diabetes of the young 5 (MODY5) due to 17q12 microdeletion with a diminished plasma glucagon level
Source: Diabetol Int. 2025 Feb 17;16(2):432–8. doi: 10.1007/s13340-025-00804-2 (PMC11954765; doi:10.1007/s13340-025-00804-2)
Supplement: Supplementary file 1 — Supplementary file1 (PPTX 46 KB) [file 13340_2025_804_MOESM1_ESM.pptx]

## Slide 1
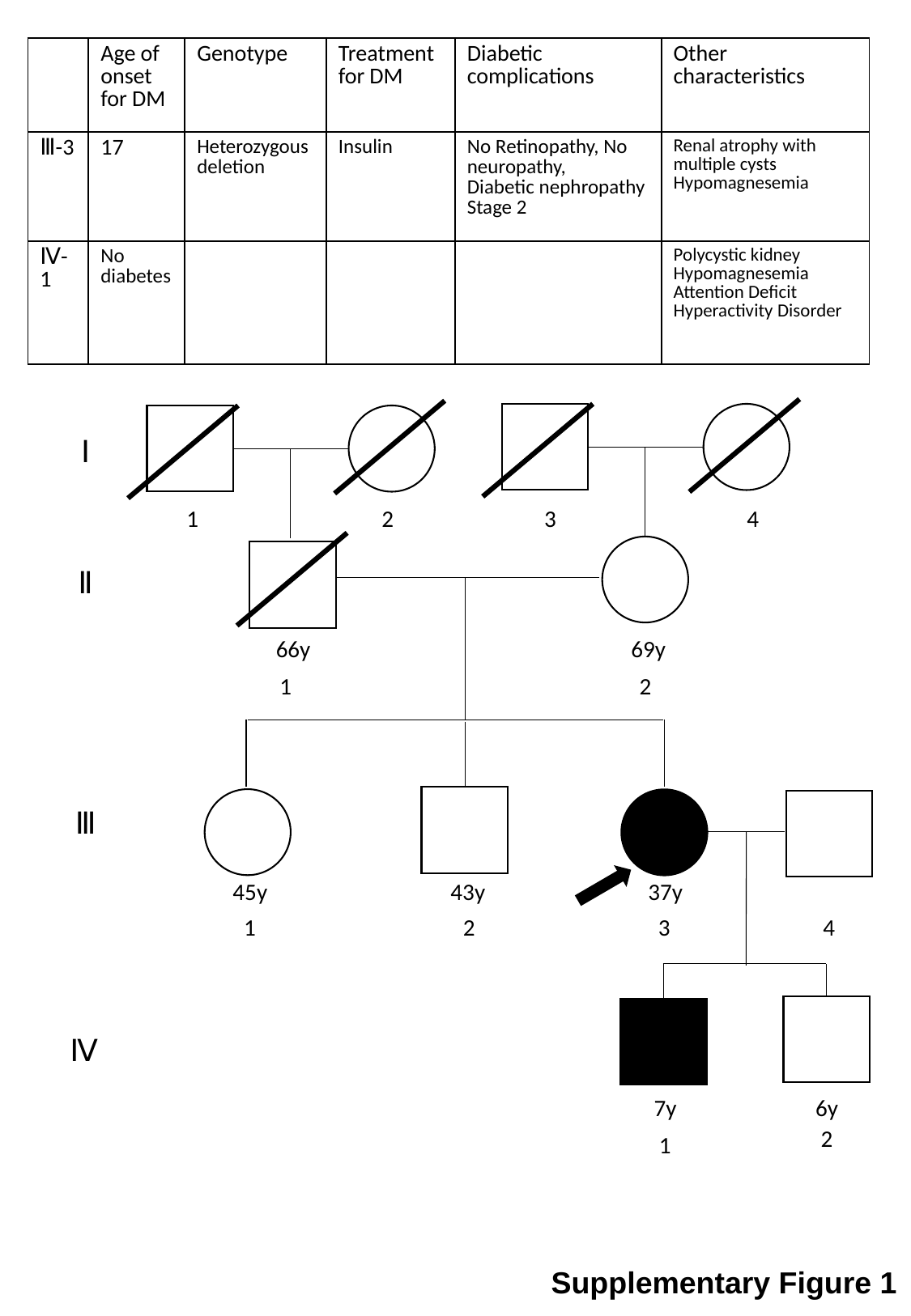

| | Age of onset for DM | Genotype | Treatment for DM | Diabetic complications | Other characteristics |
| --- | --- | --- | --- | --- | --- |
| Ⅲ-3 | 17 | Heterozygous deletion | Insulin | No Retinopathy, No neuropathy, Diabetic nephropathy Stage 2 | Renal atrophy with multiple cysts Hypomagnesemia |
| Ⅳ-1 | No diabetes | | | | Polycystic kidney Hypomagnesemia Attention Deficit Hyperactivity Disorder |
Ⅰ
1
2
3
4
Ⅱ
66y
69y
1
2
Ⅲ
45y
43y
37y
1
2
3
4
Ⅳ
7y
6y
2
1
Supplementary Figure 1
